# Mediterranean diet improves embryo yield in IVF: a prospective cohort study

**DOI:** 10.1186/s12958-019-0520-9

**Published:** 2019-09-02

**Authors:** Hongmei Sun, Yihua Lin, Dongxia Lin, Change Zou, Xiangli Zou, Lan Fu, Fanhua Meng, Weiping Qian

**Affiliations:** Department of Reproductive Center, Peking University Shenzhen Hospital, No. 1120 Lianhua Road, Shenzhen, Guangdong 518036 People’s Republic of China

**Keywords:** Mediterranean diet, Infertility, In vitro fertilization, Embryo yield

## Abstract

**Background:**

Mediterranean diet (MediDiet) had been reported to be beneficial to human health. However the relationship between diet pattern and outcomes of in vitro fertilization (IVF) treatment was scarcely researched. This study was aimed to explore the correlation between MediDiet pattern of infertile women and their clinical outcomes of IVF cycles.

**Methods:**

An observational prospective cohort study was conducted in the reproductive center from September 2016 to December 2017. Seven hundred infertile women about to undergo IVF treatment were asked to conduct a questionnaire survey. Patients were assigned to higher MediDiet adherence group or lower MediDiet adherence group according to their Mediterranean diet scores. Laboratory parameters and clinical outcomes were compared and those were different between groups were further analyzed for their relationship with MediDiet adherence.

**Results:**

A total of 590 women were finally included in the study. According to MediDiet scores, 228 participants were categorized as higher MediDiet adherence group and 362 others as lower MediDiet adherence group. No significant differences were found in baseline characteristics between groups. Higher MediDiet adherence group showed larger number of embryos available (8.40 ± 5.26 vs 7.40 ± 4.71, *P* = 0.028). Clinical pregnancy rate and implantation rate were similar between the two groups. In further correlation tests and multivariate linear regression analysis, number of fertilized oocytes and embryo yield were positively correlated to MediDiet adherence of participants.

**Conclusion:**

Infertile women with greater adherence to Mediterranean diet pattern were likely to obtain more embryos available in IVF cycle.

## Background

Infertility is becoming global challenge in both medical and social aspects. Infertile couples were reported to account for 8%~ 12% population of reproductive age and this ratio was deemed to be increasing [1]. Of the large amount of subfertile people, in vitro fertilization and embryo transfer (IVF-ET) is necessary for many to get pregnant. The outcome of IVF is correlated with a lot of factors including ovarian reserve, sperm quality, endometrial receptivity and mental stress and so on. However, as an important factor to female health, the role of dietary status in IVF treatment has been rarely studied.

There is strong evidence indicating that healthy dietary situation may be beneficial to both male and female fertility [2]. It has been reported that light dairy products [3], animal protein [4], carbohydrates [5] and trans unsaturated fats [6] are correlated with ovulatory infertility. Comparing with individual nutrients or food groups intakes, dietary pattern is considered to reflect diet habits better and influence women’s ability to conceive [7]. In a study from the USA [8], women with highest adherence to “fertility diet” (higher consumption of monounsaturated rather than trans fats, vegetable rather than animal protein sources, low carbohydrates, high fat dairy, multivitamins, and iron from plants and supplements) had a lower risk of infertility compared to women with the lowest adherence. Twigt et al. [9] found that increasing adherence to Dutch dietary recommendations in 199 women underwent IVF or intracytoplasmic sperm injection (ICSI) treatment increased the chance of ongoing pregnancy. Thus female fertility may be affected by dietary patterns, among which Mediterranean diet (MediDiet) is suspected to be potentially beneficial.

MediDiet refers to diet style of European countries on the Mediterranean coast such as Greece, France, Spain and Italy. It is characterized as diet with high intake of cereals, legumes, fruits and nuts, vegetables, fish, and olive oil but a low intake of dairy products, meat and poultry, saturated lipids, and a regularly moderate intake of alcohol [10]. Large amount of literature has proven the benefits of MediDiet to human health, including its effect of decreasing cancer rate and general mortality [11], reducing risk of obesity [12] and diabetes [13]. The update of AHA/ASA recommendations for the prevention of stroke also suggested that MediDiet could anti-hypertension and lowers the stroke risk [14].

As for the role of MediDiet on fertility, a Spanish study [15] enrolling in 2145 women of reproductive age found that the infertile risk of women with the highest quartile of adherence to MediDiet was 44% lower than those with the lowest quartile. There are only two studies concerning the relationship between MediDiet and IVF outcomes. Vujkovic et al. [16] concluded from 161 infertile couples that MediDiet by couples contribute to IVF/ICSI success rate. A recent Greece study [7] including 244 non-obese women reported significantly higher IVF clinical pregnancy rate (50.0% vs 29.1%) and live birth rate (48.8% vs 26.6%) in the highest tertile of the MediDiet score than those in the lowest tertile. Beneficial effects of MediDiet on male semen parameters have also been indicated by many researches [17–19]. However, there are a little literature reporting no relationship between female adherences to MediDiet and fertility [20].

The relationship between MediDiet and human health has been studied by numerous researches while its effect on female fertility remains scantily explored. And the only two present studies didn’t tell whether the MediDiet affect IVF outcomes through embryos or endometrium. Thus we designed a prospective cohort study with a larger sample size to explore the influence of infertile women’s adherence to MediDiet to embryo yield in IVF treatment.

## Methods

### Study population

Infertile women seeking IVF treatment in Peking University Shenzhen Hospital in Guangdong, China, from September 2016 to December 2017, were invited to participate in an ongoing prospective cohort study investigating the relationship between maternal Mediterranean dietary pattern and IVF outcome. The inclusion criteria were as follows: (1) age ≤ 40 years; (2) body mass index (BMI) ≤30 kg/m^2^; (3) use antagonist protocol or long protocol for ovarian stimulation; (4) keep dietary habit unchanged in at least recent 12 months. The study protocol was approved by the Ethics Committee of Peking University Shenzhen Hospital. Written informed consent were obtained from the participants and questionnaires were processed anonymously.

### Questionnaires and assessment of adherence to MediDiet

A food frequency questionnaire (FFQ) was designed by cooperating with specialists from the department of Nutrition in the hospital, according to the characteristics of Mediterranean dietary pattern and food composition table of China, edition 2015. The FFQ included questions about common consumed food groups (69 items regarding consumption of cereals, meat, fish, legumes, fat, fruits, nuts, vegetables, dairy products, eggs and alcoholic beverages). The frequency of food intake was expressed as times per day, times per week or times per month and the consumed amount each time were recorded as grams or milliliters.

When the patient decided to undergo IVF treatment, the FFQ was provided to and filled out by every participant to estimate the dietary pattern. The adherence to Mediterranean diet was evaluated as previously described [10] with little modification. Briefly, a score of 0 or 1 was assigned to each item of 9 components. A value of 1 was assigned when consumption was at or above the median in 5 components (cereal, legumes, vegetables, fruits and nuts, and fish) or below the median in 2 components (red meat and dairy products), and 0 was scored on the opposite situation. For fat intake, 1 score was assigned when the ratio of unsaturated lipids to saturated lipids was above the median and 0 score was assigned otherwise. There was a component about alcoholic beverage in the previous score system. Since the benefit of moderate alcohol intake to female fertility was challenged by more and more evidence [21], this item was excluded in this study. Finally, a total score range from 0 to 8 was obtained. Women with this score above the median were allocated to higher MediDiet adherence group, and those who with the score below the median were in lower MediDiet adherence group.

### IVF procedure and data extracted

IVF treatment was administrated to infertile women according to the standard operating procedure of Reproductive Center in the hospital. Ovarian reserve was tested and then protocol for controlled ovarian hyperstimulation (COH) was decided. Only those received GnRH agonist long protocol would be included. Patients were treated with 1.25 mg of Diphereline (IPSEN PHARMABIOTECH, France) on about day 21 of menstrual cycle. After the serum E2 was lower than 50 pg/ml, 75–225 IU of recombinant human follitropin (GONAL-f, Merck Serono, Switzerland) was administered according to individual response. Oocyte maturation was induced by injection of 5000–10,000 IU of HCG (Livzon Pharm, China) when the two leading follicles were 17 mm in mean diameter. Oocytes were aspirated 36 h later under the guidance of a B-ultrasound. Insemination method (IVF or ICSI) was decided according to the quality of sperm. Embryo quality was assessed on 3 days after oocyte retrieval. An embryo composed of 7–9 blastomeres and scoring with grade I or grade IIa was defined as top quality embryo. Data about female age, type of infertility, body mass index (BMI), duration of infertility, antral follicle count (AFC), basal and HCG day gonadal hormone levels, gonadotropin (Gn) duration and dosage, male age, sperm parameters, number of oocytes retrieved, insemination method, embryonic development and clinical outcomes including clinical pregnancy and implantation rate were collected for subsequent statistical analysis.

### Statistical analysis

Statistical Package for Social Science (SPSS) software, version 25.0 (IBM, Armonk, New York, USA) was used to perform all the statistical analysis. The results of continuous variables were presented as mean value ± standard deviation (SD). Categorical data were expressed as absolute frequencies and proportional rates. If continuous data complied with normal distribution, Student’s t-test was used. If not, Mann-Withney U test was applied. Categorical data were compared by a Chi-squared test. Pearson correlation test was used to estimate the correlation between adherence to MediDiet and those parameters with *P* < 0.10 in univariate analysis. A multivariate linear regression analysis was used to further estimate the influence factors on embryo yield. All reported *P* values were two tailed, and *P* < 0.05 was established as the level of significance.

## Results

A total of 700 infertile women were provided with questionnaires and 699 of them finished the FFQs, while one patient did not fulfill it. Then 21 women over 40 years old were excluded. Among the 678 patients left, 590 used Gonadotropin-releasing hormone (GnRH) agonist long protocol or GnRH antagonist protocol for COH and they were finally included for this study. They were (31.78 ± 3.72) years old in average and 53.05% (313) of them had primary infertility, 9.66% (57) had a BMI over 25 kg/m^2^. Duration of infertility was (3.43 ± 2.41) years in average.

According to individual MediDiet score, the cohort of 590 women was divided into two groups. Patients scored 0(49), 1(140), 2(173), 3(139), 4(55), 5(22) and 6(12) in total and the mean score was 2.22. Thus 228 infertile patients with a score from 3 to 6 were assigned to higher MediDiet adherence group and others were assigned to lower MediDiet adherence group. Baseline characteristics including female age, type of infertility, duration of infertility, BMI, basal gonadal hormone levels (FSH, LH, E2 and P), AFC, male age, sperm concentration and total motile sperm were all similar between the two groups as shown in Table 1.

**Table 1:** Baseline characteristics of groups with different adherence to MediDiet

	Higher adherence (*n* = 228)	Lower adherence (*n* = 362)	P
Female age (yr)	31.67 ± 3.80	31.85 ± 3.68	0.633 ^a^
Primary infertility	119 (51.19%)	194 (53.59%)	0.799 ^b^
Cause of infertility, n (%)			0.235 ^b^
Male	42 (18.4%)	93(25.7%)	
Female	85 (37.3%)	126 (34.8%)	
Combined	83 (36.4%)	118 (22.6%)	
Unknown	18 (7.9%)	25 (6.9%)	
Infertility duration (yr)	3.32 ± 2.36	3.49 ± 2.44	0.288 ^a^
BMI (kg/m^2^)	21.09 ± 2.79	21.15 ± 2.71	0.581 ^a^
Basal FSH (IU/L)	7.73 ± 2.63	7.98 ± 2.90	0.433 ^a^
Basal LH (IU/L)	5.40 ± 3.68	5.44 ± 4.20	0.921 ^a^
Basal E_2_ (pg/mL)	44.44 ± 28.67	43.53 ± 28.02	0.905 ^a^
Basal P (ng/ml)	1.17 ± 4.52	0.92 ± 2.67	0.667 ^a^
AFC	10.19 ± 5.98	10.01 ± 7.24	0.560 ^a^
Male age (yr)	34.01 ± 4.93	34.20 ± 4.86	0.715 ^a^
Sperm concentration (10^6^/mL)	56.51 ± 38.98	60.03 ± 39.23	0.371 ^a^
Total motile sperm (%)	61.65 ± 14.38	62.85 ± 14.69	0.381 ^a^

*NOTE:* Data are presented as mean ± standard deviation or n (%); yr. = year; ^a^ Mann-Withney U test; ^b^ Chi-square test

More than three quarters of patients used GnRH agonist long protocol for COH in both higher adherence group (75.88%) and lower adherence group (80.11%) and the proportions were similar between groups (*P* = 0.258). Total dosages and durations of Gn for COH were similar between groups. More oocytes were trend to be retrieved in higher MediDiet adherence group (13.98 ± 7.49) than lower adherence group (12.86 ± 6.51), but the difference was not significant. Although fertilization rates were similar in the two groups, patients in higher MediDiet adherence group (8.40 ± 5.26) had more embryos available than women with lower MediDiet adherence (7.40 ± 4.71). So far 61women in higher MediDiet adherence group and 106 others in lower MediDiet adherence group had received embryos transferred. Within similar endometrial thickness on embryo transfer day and number of embryos transferred, clinical pregnancy rate and implantation rate were also similar between the two groups. (Table 2).

**Table 2:** Comparison of IVF outcomes in groups with different MediDiet adherence

	Higher adherence (*n* = 228)	Lower adherence (*n* = 362)	P
COH protocol			0.258 ^b^
GnRH agonist long	173 (75.88%)	290 (80.11%)	
GnRH antagonist	55 (24.12%)	72 (19.89%)	
Gn duration (day)	11.60 ± 2.25	11.57 ± 2.27	0.848 ^a^
Dosage of Gn (IU)	2782.2 ± 1109.1	2840.9 ± 1160.2	0.597 ^a^
LH of HCG day (IU/L)	2.05 ± 2.37	1.95 ± 2.15	0.392 ^a^
E2 of HCG day (pg/ml)	3494.9 ± 1450.6	3303.0 ± 1396.3	0.052 ^a^
P of HCG day (ng/ml)	1.72 ± 3.18	1.57 ± 2.46	0.425 ^a^
No. of oocytes retrieved	13.98 ± 7.49	12.86 ± 6.51	0.070 ^a^
No. of fertilized oocytes	10.88 ± 6.33	9.83 ± 5.52	0.067 ^a^
Insemination method			0.754 ^b^
IVF	154 (67.54%)	240 (66.30%)	
ICSI	74 (32.46%)	122 (33.70%)	
Fertilization rate (%)	78.40 ± 19.47	76.42 ± 20.51	0.261 ^a^
No. of embryos available	8.40 ± 5.26	7.40 ± 4.71	0.028 ^a^
Available embryo rate (%)	76.32 ± 20.33	75.36 ± 21.99	0.840 ^a^
No. of top-quality embryos	2.01 ± 2.42	2.01 ± 2.47	0.967 ^a^
No. of ET cycles	61	106	
Endometrial thickness(mm)	12.69 ± 2.09	12.77 ± 2.69	0.836 ^a^
No. of embryos transferred	1.93 ± 0.25	1.99 ± 0.17	0.086 ^a^
Clinical pregnancy rate	42.62%(26)	50.94%(54)	0.300 ^b^
Implantation rate	27.97% (33/118)	31.75% (67/211)	0.533 ^b^

*NOTE:* Data are presented as mean ± standard deviation or n (%); *No.* number; *COH* controlled ovarian hyperstimulation, *Gn* gonadotropin; ^a^ Mann-Withney U test; ^b^ Chi-square test

In the Pearson correlation test, number of fertilized oocytes and embryos available were positively correlated with MediDiet adherence. To exclude potential confounding effects of some basic characteristics, the test was done again after adjusting for female age, duration of infertility and BMI. Number of fertilized oocytes and number of embryos available still showed positive correlation with MediDiet adherence (*P* = 0.039 and 0.018, respectively). E2 level in HCG day, number of oocytes retrieved were not correlated with MediDiet adherence in both tests. (Table 3).

**Table 3:** Correlation tests of MediDiet adherence and potentially correlated factors

	raw		adjusted^a^	
	r	P	adjusted-r	adjusted-P
E2 of HCG day	0.066	0.116	0.057	0.186
No. of oocytes retrieved	0.079	0.054	0.080	0.062
No. of fertilized oocytes	0.087	0.035	0.089	0.039
No. of embryos available	0.098	0.017	0.102	0.018

*NOTE:* ^a^When age, duration of infertility and BMI were adjusted; r = Pearson correlation coefficient

A multivariate linear regression analysis was then performed to explore potential confounding factors on number of embryos available. Ten variables were involved, including MediDiet adherence, female age, infertility type, duration of infertility, BMI, basal FSH, Gn duration, dosage of Gn, sperm concentration and total motile sperm. The results showed that infertility type, duration of infertility, BMI, sperm concentration and total motile sperm were not responsible for embryo yield. Instead, older age, higher basal FSH level, shorter Gn duration and smaller total dosage of Gn were risk factors for less embryos available. Interestingly, higher adherence to MediDiet was indicated to be an independent protective factor for higher embryo yield. (Table 4).

**Table 4:** Multivariate linear regression analysis of factors on number of embryos available

Variable	t	P	Unstandardized β [95% CI]	Standardized β
MediDiet adherence (a vs. b)	−2.306	0.022	−1.020 [− 1.889, − 0.151]	− 0.099
Female age	− 4.254	0.000	− 0.281 [− 0.411, − 0.151]	− 0.205
Infertility type (primary vs. secondary)	1.153	0.250	0.524 [− 0.369, 1.417]	0.052
Duration of infertility	0.511	0.610	0.046 [− 0.132, 0.225]	0.023
BMI	−0.483	0.629	−0.039 [− 0.200, 0.121]	−0.022
Basal FSH	−3.555	0.000	−0.282 [− 0.438, − 0.126]	−0.165
Gn duration	2.877	0.004	0.343 [0.109, 0.578]	0.152
Dosage of Gn	−4.516	0.000	−0.001 [− 0.002, − 0.001]	−0.259
Sperm concentration	−0.601	0.548	0.003 [−0.014, 0.008]	0.026
Total motile sperm	1.303	0.193	−0.020 [− 0.010, 0.049]	0.056

*NOTE:* β = regression coefficient; a means higher adherence to MediDiet pattern, b means lower adherence to MediDiet pattern

## Discussion

In this prospective cohort study among 700 women about to undergo IVF treatment, we assess MediDiet adherence with a professionally designed FFQ. Despite similar baseline characteristics between groups, higher adherence to MediDiet was found to be associated with more embryos available. Although E2 level in HCG day and number of oocytes retrieved seemed to be different between groups, they were not significantly correlated with MediDiet adherence in correlation tests. Clinical pregnancy rate and implantation rate were not affected by MediDiet adherence.

This study, for the first time, report that higher adherence to Mediterranean diet may improve embryo yield, while not increasing IVF success rate. This is quite different from results of previous studies. To date, only two published articles [7, 16] aimed at exploring the relationship between MediDiet and IVF outcomes. Both studies found that greater adherence to MediDiet was associated with higher pregnancy rate in IVF without improving fertilization rate and embryo yield. These results implied that the increase of IVF success rate might be attributed to improvement of endometrial receptivity instead of oocyte quality. However, as Vujkovic et al. [16] reported, preconception MediDiet elevated folate concentrations in blood and vitamin B6 level in both blood and follicular fluid. Folate [22, 23] and vitamin B6 [24] are reported to be possibly capable of improving ovarian response and oocyte quality. Furthermore, benefits of MediDiet to endometrial receptivity have not been stated by any study so far. Thus it is not convincible to conclude that MediDiet can improve endometrial receptivity.

This leads us to conjecture whether MediDiet enhances female fertility and IVF success rate by improving ovarian response and oocyte quality. When women have more and better quality oocytes, number of oocytes fertilized will also increase and as a subsequence, more embryos available will be yielded. In addition, the beneficial effect of male MediDiet adherence on semen parameters has been recognized by many published studies [17–19]. So it is substantially reasonable to speculate that the quantity and quality of oocytes may also be improved by high adherence to Mediterranean dietary pattern. This may partially explain the result of this study that higher adherence to MediDiet increase number of embryos available.

The mechanism of how MediDiet improves embryo yield remains to be a black box with presently scanty evidence. Some research clues may help to explain it. Folate level was found to ascend in both blood and follicle fluid samples of infertile women with high adherence to MediDiet [16]. It might increase number of oocyte and embryo by upregulating maturation promoting factor (MPF) through Mos-MEK-MAPK-RSK pathway [25] or by facilitating DNA methylation of oocyte and embryo [26]. Antioxidation may be another mechanism for the correlation between MediDiet and the improvement of embryo yield. The high intake of vegetables, fruit and whole grains is a character of MediDiet. Thus, this diet pattern is rich in antioxidants, more consumption of which was reported to be associated with easier conception in shorter time in patients with unexplained infertility [27]. Reactive oxygen species is known as an important cause of female infertility [28] and abundant antioxidants from MediDiet pattern may alleviate the oxidative damage on fertility. Discovery of these intrinsic mechanisms will be the highlight of further study.

However the effects of MediDiet on endometrial receptivity are not supposed to be denied now. MediDiet pattern highlights high intake of vegetable oils, most component of which is linoleic acid. As one of n-6 fatty acid molecules, linoleic acid is a precursor of prostaglandin, which is important in maintaining endometrial receptivity [16]. Hence, it is possible that MediDiet can affect implantation of embryos through regulating endometrial function. To recognize whether MediDiet affect embryos or endometrial receptivity, controlled trials can be designed in oocyte donation cycles.

In this study, clinical outcomes were not different between the two groups despite the improvement of embryo yield by MediDiet. Although number of embryos available was increased (8.40 ± 5.26 vs 7.40 ± 4.71), number of top quality embryos were similar (2.01 ± 2.42 vs 2.01 ± 2.47) between groups. This study only included fresh embryo transfer cycle to compare clinical outcomes. In most of the cycles 2 best embryos were selected to be transferred. Thus, the implantation rate was not improved. In addition, implantation of embryo needs co-working of embryo and endometrium. However the effect of MediDiet on endometrial receptivity remains unknown. So the clinical outcomes of IVF may not be significantly improved by MediDiet as well.

The results of this study indicate that Mediterranean dietary pattern may increase embryo yield through improving ovarian response or oocyte quality. During daily work of physicians in department of reproductive medicine, counselling about dietary suggestions is usually required. Comparing with active treatments such medicine and operation, diet modification seems to be more acceptable for infertile couples to enhance the possibility of getting pregnant. Outcomes of IVF are influenced by many different factors. Besides attaching importance to clinical COH strategies and laboratory manipulation of gametes and embryos, clinical workers should also pay attention to patients’ diet habits which may have tremendous potential impacts on fertility. However evidence about the relationship between diet and pregnancy is insufficient. The present study provides support for dietary counselling and reveals that diet is possibly associated with female gametes produced in IVF.

This is a prospective study with currently the largest sample size exploring the effect of MediDiet on IVF outcomes and is the first study to discover the benefits of this diet pattern of improving embryo yield. However there are some limitations for the study because only Chinese women were included and the nature of questionnaire survey may not reflect real diet situations of participants. The evaluation of MediDiet adherence was modified as deletion of the item of alcohol because the effect of ethanol on fertility has been quite controversial and only 4.9% (34) of 699 women have alcoholic intake. Next we will keep following up clinical outcomes of these patients and further analyze their relationship with MediDiet adherence.

## Conclusion

In conclusion, this study indicates that higher adherence to Mediterranean dietary pattern of infertile women may improve embryo yield in IVF treatment cycle. More well-designed studies are needed to verify our result and further validate the effect of Mediterranean diet on female fertility and IVF outcomes.

## Data Availability

The datasets used and/or analyzed during the current study available from the corresponding author on reasonable request.

## Abbreviations

AFC: Antral follicle count
BMI: Body mass index
COH: Controlled ovarian hyperstimulation
FFQ: Food frequency questionnaire
Gn: Gonadotropin
GnRH: Gonadotropin-releasing hormone
HCG: Human chorionic gonadotropin
ICSI: Intracytoplasmic sperm injection
IVF: In vitro fertilization
MediDiet: Mediterranean diet
MPF: Maturation promoting factor
